# Vitamin C Deficiency Masquerading as Vasculitis in a Patient With Crohn’s Disease

**DOI:** 10.7759/cureus.55295

**Published:** 2024-02-29

**Authors:** Kritin K Verma, Fatma Z Deligonul, Helen Chen, Michelle Tarbox

**Affiliations:** 1 School of Medicine, Texas Tech University Health Sciences Center, Lubbock, USA; 2 School of Osteopathic Medicine, University of Incarnate Word, San Antonio, USA; 3 Dermatology, Texas Tech University Health Sciences Center, Lubbock, USA

**Keywords:** histopathology (hp), crohn's disease, vasculitis, vitamin c deficiency, scurvy

## Abstract

This case study features a 40-year-old male with Crohn's disease (CD) who was initially misdiagnosed with vasculitis but was later shown to have scurvy owing to vitamin C deficiency. The patient's diet was nearly exclusively made up of highly processed fast food, with no fresh fruits or vegetables. A mildly sensitive, violaceous rash on his lower legs, mild gingival hemorrhage and enlargement, and muscle soreness were among his symptoms. Anemia and undetectable vitamin C levels were discovered in laboratory studies. A skin sample revealed follicular hyperkeratosis, coiled hairs, and perifollicular bleeding, eliminating the possibility of vasculitis. Scurvy was confirmed by undetectable vitamin C levels and intramuscular bleeding discovered during a muscle biopsy. After one month of vitamin C administration, the patient's skin was entirely clear. This instance emphasizes the significance of taking vitamin C insufficiency into account in patients with CD and other disorders that can cause malabsorption. Misdiagnosis might result in unneeded treatments and medical expenses. Scurvy must be diagnosed as soon as possible because it might cause gastrointestinal/intracerebral hemorrhage and death.

## Introduction

Vitamin C deficiency is an uncommon but potentially fatal illness that can manifest as a range of symptoms, as it is required for the manufacturing of collagen and the hydroxylation of proline residues on procollagen, which allows hydrogen bonding to create its fundamental triple-helix form. Vitamin C can be obtained from the diet, mainly from fruits and vegetables. Absorption occurs in the distal small bowel, and body stockpiles might be depleted after one to three months [1]. Malabsorption due to inflammatory bowel diseases like Crohn’s disease (CD), medications, increased metabolic demand, and inadequate intake are all potential causes of vitamin C deficiency [2]. In this case report, we present a patient with CD who was discovered to have scurvy, an illness caused by a lack of vitamin C, which was previously misdiagnosed as vasculitis.

## Case presentation

**Figure 1:**
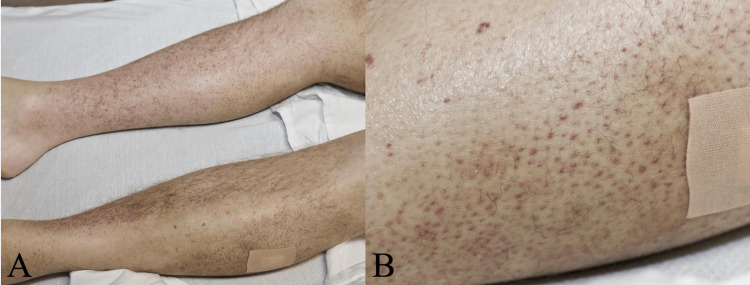
Distant (A) and close-up (B) presentation of the patient’s onset of corkscrew-like hair with hyperkeratotic papules with perifollicular hemorrhage and petechiae

A 40-year-old male with an 8-year history of CD and a diverting ileostomy was admitted to the hospital with a two-week history of a mildly tender, violaceous folliculocentric eruption on his lower extremities and was misdiagnosed as vasculitis (Figures 1A, 1B).

**Figure 2:**
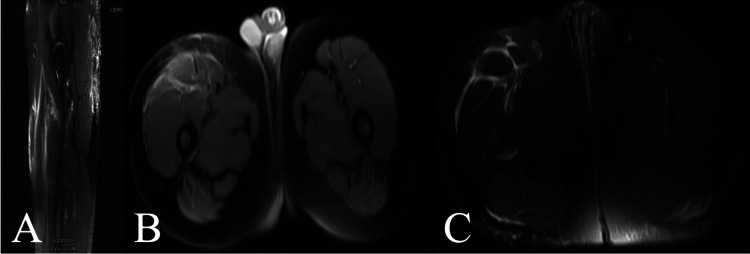
MRI showing patchy, edema-like signal throughout the right rectus femoris from the hip to the knee Frontal (A), transverse T1 (B), and transverse T2 (C) views

He also reported mild gingival bleeding and hypertrophy. Upon further questions, he revealed his diet, which was almost entirely heavily processed fast food and had no sources of fresh fruits or vegetables. His vitamin C level was undetectable, and his hemoglobin and hematocrit were 7.9 g/dL and 24.7%, respectively. The patient complained of muscle pain in bilateral legs. Upon physical examination, a painful indurated plaque was noted on his thigh, which prompted imaging with an MRI to rule out other causes like malignancy or infection. MRI findings were suggestive of intramuscular hemorrhage (Figures 2A-2C). A muscle biopsy was performed later to rule out other sources of sepsis or malignancy.

**Figure 3:**
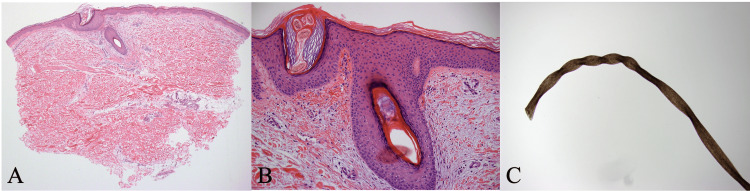
Skin biopsy of the patient’s thigh demonstrating perifollicular hemorrhage and absence of inflammation H&E 100x magnification (A), H&E 200x magnification (B) Vitamin C deficiency leading to improper collagen synthesis and ca​​using structural deformation of the hair follicle (C)

Then, a skin biopsy was performed and showed follicular hyperkeratosis, coiled hairs, and perifollicular hemorrhage in the absence of inflammation, which made vasculitis unlikely to be the cause of his rash (Figures 3A-3C).

**Figure 4:**
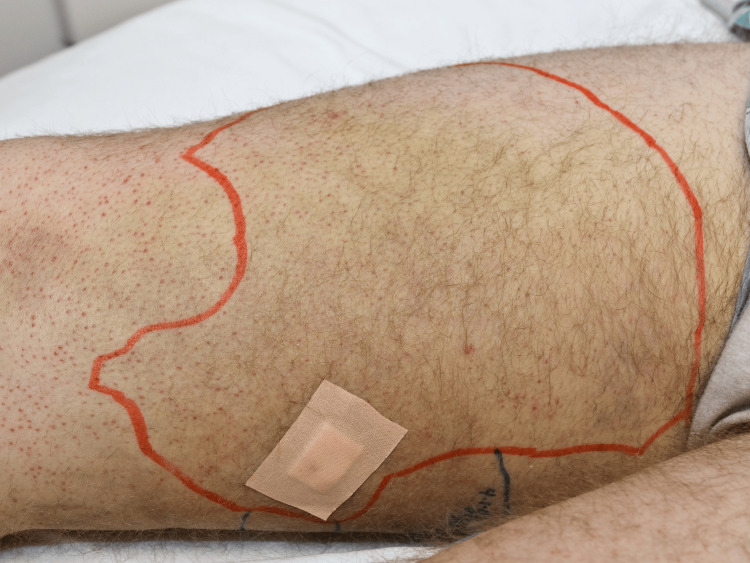
Firm and indurated plaque demarcated by a red line on the right thigh with the site of muscle biopsy at the lower left corner

The diagnosis of scurvy was further supported by undetectable vitamin C levels as well as intramuscular hemorrhage found on muscle biopsy (Figure 4).

**Figure 5:**
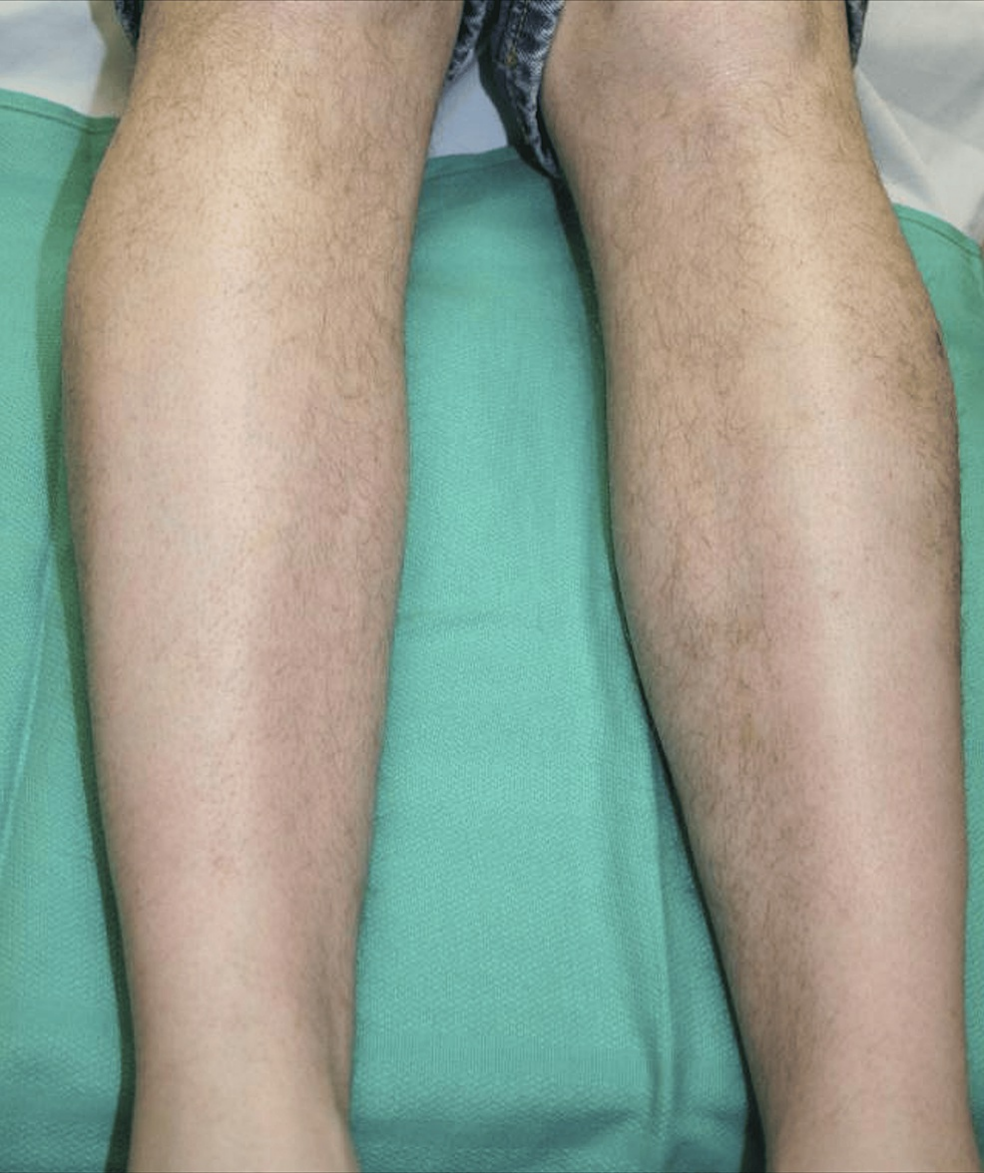
Resolution of the patient’s petechial rash on bilateral lower legs after one month of vitamin C treatment

The patient was treated with 1,000 mg of vitamin C daily for one week, followed by 100 mg daily thereafter. The patient’s skin completely cleared after one month of vitamin C supplementation (Figure 5).

## Discussion

In this case, the patient’s scurvy was initially misdiagnosed as vasculitis, highlighting the importance of considering vitamin C deficiency in patients with CD and other conditions that can lead to malabsorption. Vitamin C deficiency can be caused by malabsorption (due to conditions affecting the gastrointestinal tract such as CD), increased loss or requirement, and inadequate intake [1]. Patients at increased risk for vitamin C deficiency include the elderly; those taking medications such as indomethacin, oral contraceptives, tetracyclines, or corticosteroids; patients on a restricted diet secondary to inflammatory bowel disease; and patients with alcohol use disorder, anorexia, cancer, or renal failure [1,2]. Vitamin C deficiency can lead to significant signs and symptoms, including fatigue, depression, and connective tissue defects such as gingivitis, erythematous rashes, petechiae, periosteal and perifollicular hemorrhage, and impaired wound healing [1,2]. In infants and children, bone growth is impaired, and bleeding and anemia may also occur [1].

Diagnosis of vitamin C deficiency is usually made clinically in a patient who has skin or gingival signs and is at risk of vitamin C deficiency [1]. A complete blood count (CBC) is obtained to help determine anemia. Bleeding, bruising, and petechiae are common findings in patients with scurvy [1]. Measuring the vitamin C level in the blood can also help establish the diagnosis [3]. Scurvy can easily be treated by incorporating more vitamin C into the diet, such as fresh fruit and vegetables, and through supplementation [1,2]. Most patients with scurvy treated with vitamin C supplements feel better within 48 hours and make a full recovery within two weeks [4].

The diagnosis of scurvy in patients with concomitant GI issues, such as inflammatory bowel disease (IBD) or CD, has been observed to be difficult. A case report revealed a pediatric patient who presented with symptoms suggestive of scurvy to later be diagnosed with persistent non-infectious osteomyelitis, which was the patient’s first presenting symptom of CD [5]. Another case report described a patient with perifollicular petechiae of the limbs, a severe lower extremity hematoma, and sacral osteopenia who was misdiagnosed with leukocytoclastic vasculitis on multiple occasions [6]. A study indicated that 21.6% of IBD patients, including 24.4% of CD patients, lacked vitamin C [2]. This is notable in comparison to the general population, where vitamin C insufficiency is significantly less common: in high-income countries, the prevalence of vitamin C deficiency ranges from 2.2% to 5.7% in men and presents at an even lower rate in women [7]. Additionally, vitamin C insufficiency may be more difficult to detect in CD patients because their symptoms of active CD may overlap with those of scurvy [8]. These cases emphasize the significance of considering vitamin C deficiency among other cutaneous manifestations of malabsorption in patients with IBD, such as angular cheilitis (vitamin B), pellagra (niacin), and acrodermatitis enteropathica (zinc), as well as the difficulties in identifying scurvy in these types of patients [9].

## Conclusions

It is important to diagnose scurvy early, as it can lead to GI/intracerebral hemorrhage and death. Misdiagnosis can lead to unnecessary procedures and additional medical expenses. In this case, the patient’s scurvy was initially misdiagnosed as vasculitis, highlighting the importance of considering vitamin C deficiency in patients with Crohn’s disease and other conditions that can lead to malabsorption.
